# The burden of osteoarthritis due to high Body Mass Index in Iran from 1990 to 2019

**DOI:** 10.1038/s41598-023-37780-z

**Published:** 2023-07-20

**Authors:** Mitra Darbandi, Fatemeh Khosravi Shadmani, Mahsa Miryan, Mojtaba Ghalandari, Mahsa Mohebi, Samira Arbabi Jam, Yahya Pasdar

**Affiliations:** 1 Research Center for Environmental Determinants of Health (RCEDH), Health Institute, Kermanshah University of Medical Sciences, Kermanshah, Iran; 2 Nutritional Sciences Department, School of Nutritional Sciences and Food Technology, Kermanshah University of Medical Sciences, Kermanshah, Iran; 3 Zanjan University of Medical Sciences, Zanjan, Iran; 4 Student Research Committee, Kermanshah University of Medical Sciences, Kermanshah, Iran; 5 Cardiovascular Research Center, Kermanshah University of Medical Sciences, Kermanshah, Iran; 6 Nutritional Sciences Department, School of Nutritional Sciences and Food Technology, Isar Square, Kermanshah, Iran

**Keywords:** Health care, Medical research

## Abstract

High BMI related burden of knee and hip osteoarthritis (OA) is on a significant rise worldwide. OA not only causes joint pain and stiffness, but it also leads to disability. This study investigated the trend and burden of OA attributable to high body mass index (BMI) in Iran. The age-standardized disability-adjusted life years (DALYs) rates of knee and hip OA due to high BMI, were estimated using data from the Global Burden of Disease 2019. We evaluated DALYs rate trend of high BMI related OA by sex and age in span of 30 years from 1990–2019 across the 31 provinces of Iran. The age-standardized prevalence trend of OA in the knee and hip showed an increase from 1990 to 2019. In 2019 there were 29.92 (95% CI: 10.98–64.92) and 42.50 (95% CI: 16.32–97.37) DALYs/100,000 related to OA from high BMI in men and women, respectively. 2019 saw the greatest DALYs/100,000 rate in the 65–79 age group. From 2005 to 2019, men and women saw DALYs/100,000 rate changes of 24.87 and 17.43 percent, respectively. The burden of knee OA was significantly higher than that of hip OA. DALYs rate of OA due to high BMI was found to be positively associated with the Socio-demographic Index (SDI). The burden of knee and hip OA due to high BMI has increased significantly in recent years in Iran among all age groups of both men and women. It is recommended that health policymakers develop weight control strategies to reduce the burden of OA and implement them at the national level**.**

## Introduction

Osteoarthritis (OA) is the most common musculoskeletal condition is. Pain, loss of function, and occasional stiff joints are the major side effects of OA^[1,2]^. The incidence of OA has recently increased due to aging, obesity, and the advent of some of the new sports^[3–5]^. Also, the burden of high body mass index (BMI), an important factor of disability-adjusted life years (DALYs), has grown significantly in recent years^[6–8]^. Scientific evidence shows that the burden of OA caused by high BMI has grown in recent decades as well^[4,9,10]^. Results from a population-based cohort study have shown that high BMI increases the risk of knee and hip OA by 53% in women (hazard ratio: 1.53) and 20% in men (hazard ratio: 1.20), with a dose–response relationship and a stronger risk with increasing BMI^[11]^. Two potential processes, an increase in mechanical pressure on joints and the emergence of inflammation, explain the occurrence of OA from overweight or obesity^[12–14]^. Inflammation occurs and intensifies in OA patients as a result of obesity and the associated rise in adipose tissue, which creates a milieu of low-grade systemic inflammation. Increased levels of adipokines (leptin, adiponectin) and pro-inflammatory cytokines, such as tumour necrosis factor-alpha (TNF-a), interleukin-6 (IL-6), and IL-1, which have been linked to OA, are secreted from the adipose tissue of obese people^[15,16]^. individuals with OA have abnormally elevated levels of TNF-, IL-6, and IL-1, which have been identified as a key contributor to cartilage degradation^[15,17]^.

To date, no comprehensive study has been conducted to investigate the burden of knee and hip OA attributed to high BMI in Iran. Therefore, the aim of this study was to determine the prevalence and DALYs of OA attributable to high BMI and the distribution among provinces from 1990 to 2019 by sex and age. The results of this study provide important insights for prioritizing and accurate planning of health services in order to control OA.

## Methods

### Overview

The Institute of Health Metrics and Evaluation (IHME) undertook the biggest worldwide observational epidemiological study to date, the global Burden of Disease 2019 Study (GBD 2019). GBD research has included 175 nations and territories, 7 super-regions, and 21 regions since 1990. As of 2017 (https://vizhub.healthdata.org/gbd-compare/), 359 illnesses and injuries, 282 causes of death, and 84 risks have been thoroughly researched.

### Data source and case definition

In the present study, to determine the prevalence and burden of obesity-related osteoarthritis in the Iranian provinces, we extracted obesity-related osteoarthritis data from the GBD by age and sex in the last three decades.

Only osteoarthritis (OA) of the hip and knee was included in the GBD 2017 Study, with symptomatic OA of the hip or knee validated radiologically as Kellgren-Lawrence grade 2–4 as the reference definition^[18,19]^. A distinct osteophyte in the hip or knee, together with discomfort for at least one month over the previous 12 months, are indicators of symptomatic grade 2 OA. Osteophytes and a narrowing of the hip or knee joint space are signs of symptomatic grade 3–4 OA, while deformity and discomfort lasting at least one month during the previous 12 months are signs of grade 4 OA^[18,19]^. Hip and knee joints are often affected by OA in bigger joints and are thought to cause the most impairment. Therefore, only these joints were taken into consideration to get GBD OA estimations. An expensive joint replacement may be required if these joints fail, which would result in a rise in the direct cost of healthcare. BMI ≥ 25 kg/m^2^, defined as high BMI in the GBD 2019 study, was introduced as a risk factor for OA.

In this study, data were extracted separately for men and women from 1990 to 2019 and for 13 age groups from 30 to 94 years. Iran has a total of 31 provinces and all provinces were included in this study. Therefore, we extracted data for 31 provinces, including West Azarbaijan, Yazd and Zanjan, South Khorasan, Ardebil, East Azarbaijan, Esfahan, Fars, Hormozgan, Kerman, Khuzestan, Buyer Ahmad, Kohgiluyeh, Alborz, Bushehr, Chahar Mahall and Bakhtiari, Gilan, Golestan, Hamadan, Ilam, and Kermanshah.

We determined the age-standardized prevalence rate (per 100,000 population) for knee and hip osteoarthritis in all the 31 provinces. In addition, data on knee and hip DALYs attributable to high BMI in the provinces were obtained using the Global Health Data Exchange search tool. (https://vizhub.healthdata.org/gbd-compare/).

DALY was computed as follows: DALY = Years lost due to disability (YLD) + years of life lost (YLL). It should be highlighted that there was no proof of cause-specific death linked to OA in the GBD 2019 study; and DALYs for OA was calculated without taking mortality into account. Therefore, the values discovered for YLD and DALYs are nearly identical to those for OA.

### Statistical analysis

Age-standardized DALYs were used to assess the variations in the burden of knee and hip OA by era and sex in order to control discrepancies in the age composition of the population. We present age-standardized estimates for DALYs rates with 95% uncertainty interval (lower, upper) and the percentage of 10-year change from 1990 to 2019 at national and provincial levels. Rates were expressed per 100,000 persons. Three time points were used to compute percentage changes by sex and province. R software 4.0.2 (2020.06.22) was also used for all figures.

### Ethics approval and consent to participate

The study was approved by the ethics committee of Kermanshah University of Medical Sciences (KUMS.REC.1401.238). All methods were carried out by relevant guidelines and regulations.

## Results

### Trends in the prevalence of OA from 1990 to 2019

**Figure 1:**
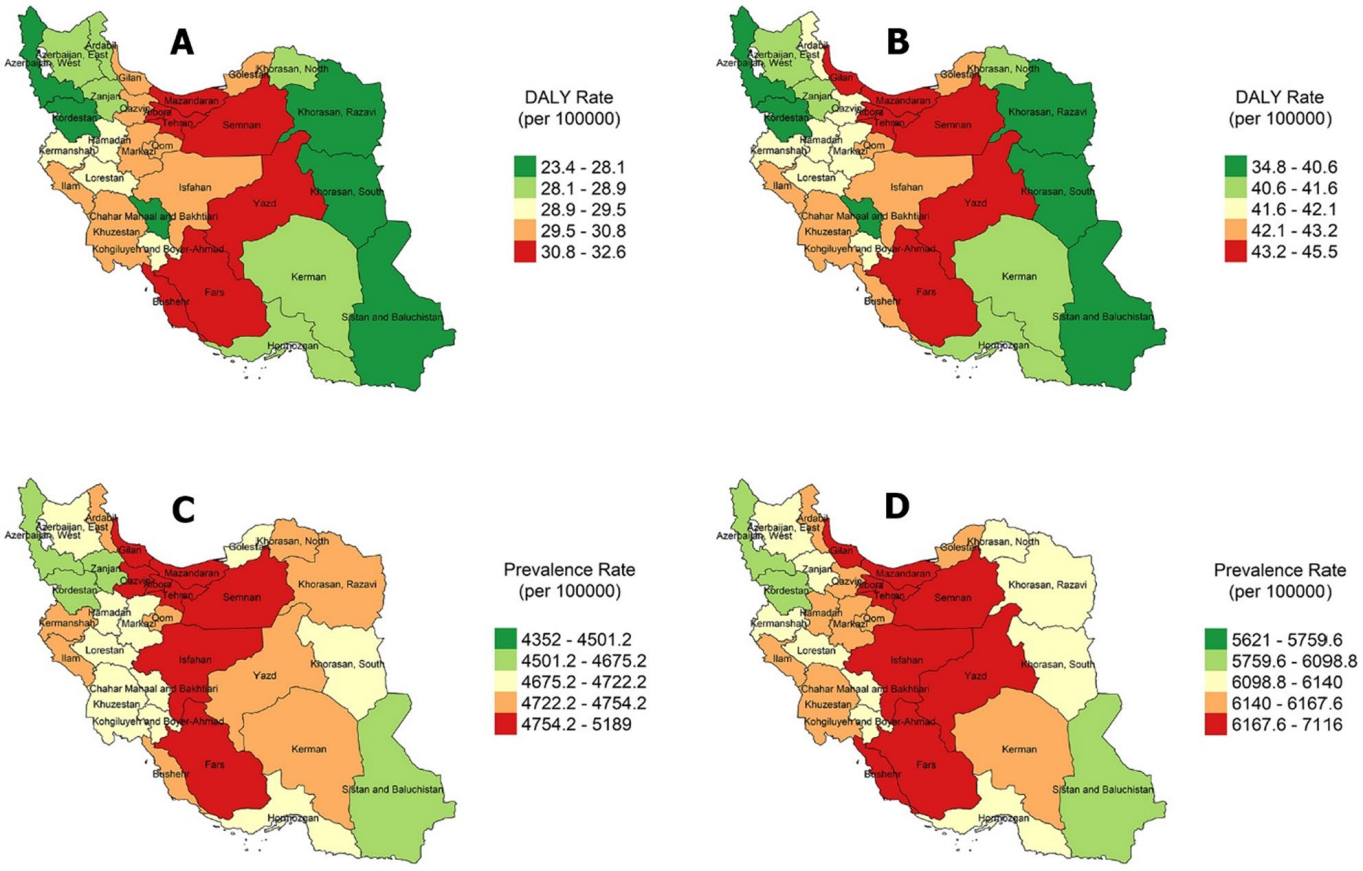
Trends of DALYs rate (per 100,000 population) in men (**A**) and women (**B**) and Prevalence rate (per 100,000 population) Osteoarthritis attributable to high BMI in men (**C**) and women (**D**) in Iran (2019).

Trends in the age-standardized prevalence of OA due to high BMI from 1990 to 2019 in 31 provinces of Iran by sex are shown in Table 1. The prevalence of OA due to high BMI in Iranian men and women in 1990 was 4575 and 5916.39 per 100,000 people, respectively. The prevalence of high BMI related OA in Iranian men and women in 2019 was 4836.22 and 6337.48 per 100,000 people, respectively. Age-standardized prevalence of OA rose between 1990 and 2005, as indicated in the table, with percentage increases of 4.49 and 5.55 for men and women, respectively. For males and femals, respectively, the changes in the prevalence of OA from 2005 to 2019 were 1.16 and 1.47. The increase from 1990 to 2005 was much higher than the increase from 2005 to 2019, with the largest increase in North Khorasan, South Khorasan, Kohgiluyeh and Boyer-Ahmad, and Tehran from 2005 to 2019 (Fig. 1C,D).

**Table 1:** Age-Standardized prevalence rate (per 100,000 population) with 95% uncertainty interval (lower, upper) of knee and hip osteoarthritis attributable to high BMI, the Global Burden of Disease 2019 Study.

Province	Prevalence (Per 100,000 population)						Percent change (%)			
	1990		2005		2019		1990 – 2005		2005 – 2019	
	Men	Women	Men	Women	Men	Women	Men	Women	Men	Women
Alborz	4631.97 (4132.79 – 5147.97)	5884.15 (5234.46 – 6625.77)	4806.48 (4302.91 – 5358.42)	6230.39 (5552.97 – 7025.36)	4826.17 (4313.78 – 5382.70)	6235.36 (5566.83 – 6977.63)	3.76	5.88	0.40	0.07
Ardebil	4451.23 (3996.77 – 4965.24)	5714.34 (5073.69 – 6424.58)	4698.48 (4216.62 – 5224.05)	6135.90 (5448.55 – 6940.35)	4735.57 (4237.42 – 5247.52)	6144.02 (5489.29 – 6899.79)	5.55	7.37	0.78	0.13
East Azarbayejan	4477.63 (4018.23 – 4942.78)	5735.68 (5095.77 – 6452.84)	4693.22 (4195.65 – 5244.95)	6125.13 (5467.37 – 6942.95)	4735.30 (4220.29 – 5272.18)	6140.14 (5481.36 – 6869.43)	4.81	6.78	0.89	0.24
West Azarbayejan	4430.24 (3973.22 – 4944.66)	5689.20 (5068.06 – 6426.13)	4654.13 (4173.55 – 5170.57)	6081.90 (5413.44 – 6900.88)	4676.34 (4199.45 – 5190.81)	6093.15 (5415.44 – 6874.75)	5.05	6.90	0.47	0..18
Bushehr	4518.40 (4030.83 – 5021.83)	5771.27 (5119.74 – 6518.45)	4744.24 (4240.73 – 5304.78)	6167.53 (5506.15 – 6931.11)	4777.27 (4261.96 – 5321.36)	6185.76 (5516.74 – 6928.60)	4.99	6.86	0.69	0.29
Chahar Mahaal and Bakhtiari	4454.82 (3964.73 – 4967.53)	5713.27 (5087.13 – 6433.10)	4670.02 (4187.51 – 5199.78)	6092.62 (5429.19 – 6897.41)	4706.22 (4205.98 – 5230.04)	6099.51 (5444.13 – 6851.84)	4.83	6.63	0.77	0.11
Fars	4521.07 (4058.47 – 5009.14)	5780.83 (5181.02 – 6524.28)	4755.65 (4257.98 – 5289.11)	6182.28 (5526.25 – 6977.01)	4784.29 (4282.51 – 5290.48)	6192.84 (5511.63 – 6944.15)	5.18	6.94	0.60	0.17
Gilan	4544.42 (4057.52 – 5053.00)	5784.97 (5163.13 – 6521.92)	4763.89 (4263.01 – 5296.85)	6183.55 (5516.77 – 6983.88)	4788.77 (4294.05 – 5322.60)	6196.18 (5534.98 – 6960.26)	4.82	6.88	0.52	0.20
Golestan	4506.26 (4035.54 – 5016.65)	5766.79 (5122.90 – 6523.72)	4722.31 (4243.04 – 5234.07)	6144.61 (5476.88 – 6948.10)	4756.48 (4261.04 – 5279.05)	6156.59 (5517.64 – 6919.91)	4.79	6.55	0.72	0.19
Hamadan	4493.64 (4000.81 – 4981.89)	5749.40 (5157.08 – 6487.23)	4721.70 (4223.86 – 5277.06)	6155.42 (5500.72 – 6933.60)	4759.72 (4264.42 – 5301.04)	6161.50 (5511.48 – 6912.88)	5.07	7.06	0.80	0.09
Hormozgan	4452.07 (3989.67 – 4965.69)	5701.40 (5093.23 – 6417.61)	4698.31 (4199.65 – 5245.92)	6119.82 (5451.42 – 6919.85)	4740.20 (4250.39 – 5249.52)	6137.31 (5495.36 – 6864.69)	5.53	7.33	0.89	0.28
Ilam	4495.49 (4035.44 – 5018.46)	5742.60 (5118.59 – 6505.15)	4739.45 (4244.19 – 5251.81)	6167.44 (5518.22 – 6963.53)	4772.45 (4275.17 – 5276.85)	6180.67 (5549.08 – 6932.39)	5.42	7.39	0.69	0.21
Isfahan	4554.72 (4079.82 – 5037.79)	5803.38 (5179.55 – 6530.39)	4752.72 (4262.81 – 5291.02)	6177.45 (5535.13 – 6966.31)	4774.81 (4300.85 – 5270.58)	6186.06 (5523.56 – 6910.42)	4.34	6.44	0.46	0.13
Kerman	4532.89 (4047.52 – 5022.07)	5791.74 (5148.74 – 6538.75)	4723.00 (4237.23 – 5260.33)	6158.98 (5487.38 – 6957.78)	4737.56 (4232.49 – 5260.88)	6140.75 (5481.41 – 6941.16)	4.19	6.34	0.30	– 0.29
Kermanshah	4473.91 (4006.53 – 4984.64)	5736.00 (5119.66 – 6456.04)	4690.91 (4210.01 – 5212.52)	6114.99 (5448.61 – 6916.14)	4734.65 (4249.80 – 5252.74)	6132.02 (5482.41 – 6903.96)	4.85	6.60	0.93	0.27
North Khorasan	4423.95 (3966.87 – 4906.60)	5692.80 (5047.14 – 6360.26)	4678.30 (4194.54 – 5250.99)	6112.84 (5454.07 – 6922.12)	4737.87 (4250.28 – 5288.80)	6136.47 (5492.67 – 6950.31)	5.74	7.37	1.27	0.38
Khorasan – e – Razavi	4488.17 (4016.42 – 4996.20)	5743.46 (5127.67 – 6461.97)	4707.20 (4219.58 – 5210.68)	6124.29 (5447.21 – 6926.64)	4726.75 (4223.76 – 5238.20)	6126.79 (5492.23 – 6860.17)	4.88	6.63	0.41	0.04
South Khorasan	4425.73 (3940.85 – 4916.61)	5687.72 (5077.81 – 6418.13)	4662.95 (4173.31 – 5192.99)	6097.69 (5437.61 – 6912.52)	4716.01 (4228.39 – 5253.15)	6115.91 (5475.81 – 6844.89)	5.36	7.20	1.13	0.29
Khuzestan	4485.91 (3994.56 – 4996.17)	5735.96 (5126.70 – 6484.41)	4712.24 (4209.66 – 5243.91)	6141.55 (5490.53 – 6937.72)	4747.40 (4235.99 – 5252.92)	6150.58 (5509.24 – 6888.89)	5.04	7.07	0.74	0.14
Kohgiluyeh and Boyer – Ahmad	4449.23 (3974.71 – 4947.33)	5697.57 (5091.90 – 6429.99)	4691.40 (4213.58 – 5239.67)	6117.20 (5472.86 – 6937.49)	4742.51 (4236.65 – 5266.63)	6144.16 (5487.93 – 6925.83)	5.44	7.36	1.08	0.44
Kurdistan	4408.69 (3934.93 – 4891.03)	5678.14 (5042.18 – 6442.86)	4654.84 (4169.12 – 5192.67)	6077.37 (5440.52 – 6901.67)	4694.68 (4200.17 – 5202.20)	6098.91 (5449.07 – 6851.02)	5.58	7.03	0.85	0.35
Lorestan	4477.91 (4011.01 – 4983.68)	5728.96 (5100.00 – 6458.60)	4710.67 (4226.88 – 5221.20)	6130.31 (5452.12 – 6940.50)	4743.93 (4255.03 – 5266.52)	6147.90 (5507.35 – 6147.90)	5.19	7.00	0.70	0.28
Markazi	4498.41 (4011.44 – 5018.94)	5756.31 (5141.90 – 6482.91)	4716.21 (4214.30 – 5234.81)	6140.26 (5476.61 – 6912.21)	4741.40 (4242.04 – 5258.14)	6147.42 (5497.65 – 6904.48)	4.84	6.67	0.53	0.11
Mazandaran	4555.20 (4066.01 – 5078.63)	5802.25 (5167.03 – 6502.93)	4785.63 (4291.47 – 5307.43)	6196.10 (5516.05 – 6988.40)	4830.23 (4333.61 – 5358.84)	6222.02 (5555.11 – 6977.89)	5.05	6.78	0.93	0.41
Qazvin	4513.46 (4039.25 – 5062.77)	5764.97 (5122.56 – 6514.04)	4713.86 (4224.49 – 5239.42)	6143.43 (5475.04 – 6969.10)	4755.26 (4263.85 – 5297.34)	6159.55 (5503.52 – 6915.27)	4.44	6.56	0.87	0.26
Qom	4495.13 (4013.41 – 4978.01)	5748.54 (5135.08 – 6466.28)	4707.79 (4216.23 – 5231.55)	6131.58 (5488.31 – 6940.09)	4744.14 (4257.77 – 5244.42)	6152.91 (5504.23 – 6926.00)	4.73	6.66	0.77	0.34
Semnan	4515.31 (4028.21 – 5025.23)	5766.38 (5123.49 – 6491.44)	4742.35 (4246.33 – 5297.09)	6167.20 (5505.30 – 6978.48)	4770.05 (4261.56 – 5287.71)	6180.02 (5531.24 – 6970.13)	5.02	6.95	0.03	0.20
Sistan and Baluchistan	4351.96 (3891.77 – 4843.70)	5620.90 (5018.84 – 6355.23)	4575.62 (4115.44 – 5108.67)	6012.05 (5361.70 – 6820.60)	4615.46 (4110.29 – 5131.57)	6014.03 (5389.20 – 6735.24)	5.13	6.95	0.87	0.03
Tehran	4994.28 (4486.91 – 5545.30)	6765.75 (5986.05 – 7670.72)	5066.84 (4549.07 – 5636.14)	6730.35 (6051.01 – 7550.51)	5188.75 (4666.52 – 5758.88)	7115.85 (6340.33 – 8022.47)	1.45	− 0.52	2.40	5.72
Yazd	4537.86 (4062.57 – 5053.58)	5790.01 (5155.50 – 6525.44)	4743.32 (4235.59 – 5277.56)	6171.46 (5505.38 – 6970.69)	4790.58 (4273.62 – 5328.93)	6192.14 (5541.46 – 7003.14)	4.52	6.58	0.99	0.33
Zanjan	4457.00 (3983.93 – 4959.88)	5721.74 (5094.03 – 6439.00)	4671.52 (4186.53 – 5182.18)	6100.15 (5455.06 – 6883.47)	4715.69 (4226.22 – 5215.07)	6123.77 (5471.98 – 6884.22)	4.81	6.61	0.94	0.38
Iran	4575.00 (4118.27 – 5069.33)	5916.39 (5303.04 – 6656.70)	4780.62 (4298.90 – 5286.34)	6245.26 (5591.40 – 7037.53)	4836.22 (4354.14 – 5353.03)	6337.48 (5706.85 – 7098.65)	4.49	5.55	1.16	1.47

### Trends in DALYs rates for OA due to high BMI from 1990 to 2019

The trend in age-standardized DALY rates of OA attributed to high BMI in 31 Iranian provinces is shown from 1990 to 2019 by sex in Table 2. In Iran, the DALYs rates of OA related to high BMI have increased significantly for both sexes between 1990 and 2019.

**Table 2:** Age-Standardized DALYs rate (per 100,000 population) with 95% uncertainty interval (lower, upper) of knee and hip osteoarthritis attributable to high BMI, the Global Burden of Disease 2019 Study.

Province	DALYs (Per 100,000 population)						Percent change (%)			
	1990		2005		2019		1990 – 2005		2005 – 2019	
	Men	Women	Men	Women	Men	Women	Men	Women	Men	Women
Alborz	18.00 (5.81 – 41.5)	31.07 (11.05 – 71.95)	25.30 (8.98 – 54.58)	38.32 (14.29 – 87.29)	31.46 (11.60 – 68.94)	44.60 (16.98 – 100.53)	40.55	23.33	24.34	16.38
Ardebil	14.21 (4.42 – 34.35)	26.56 (9.10 – 62.27)	22.86 (8.00 – 50.95)	35.67 (13.25 – 80.93)	28.70 (10.01 – 61.68)	41.88 (15.57 – 95.89)	60.87	34.29	25.54	17.40
East Azarbayejan	15.47 (4.84 – 36.39)	27.19 (9.63 – 63.84)	22.77 (7.90 – 49.95)	34.86 (12.70 – 80.20)	28.56 (10.34 – 62.63)	41.24 (15.43 – 94.93)	47.18	28.20	25.42	18.30
West Azarbayejan	13.89 (4.30 – 31.83)	25.65 (8.66 – 58.74)	20.93 (7.00 – 45.88)	33.13 (12.03 – 77.74)	25.95 (9.25 – 55.81)	38.74 (14.27 – 88.94)	50.68	29.16	23.98	16.93
Bushehr	15.96 (4.94 – 37.03)	27.61 (9.44 – 64.76)	24.71 (8.99 – 53.46)	36.45 (13.65 – 81.83)	31.01 (11.57 – 67.82)	43.08 (16.21 – 95.48)	54.82	32.01	25.65	18.18
Chahar Mahaal and Bakhtiari	11.54 (3.22 – 27.49)	22.19 (7.31 – 52.48)	18.12 (5.92 – 41.00)	29.72 (10.37 – 70.78)	23.67 (8.10 – 53.01)	35.64 (12.85 – 83.21)	57.01	33.93	30.62	19.91
Fars	16.28 (5.16 – 36.92)	28.23 (9.88 – 65.53)	26.01 (9.21 – 57.62)	38.14 (14.41 – 84.80)	31.53 (11.76 – 68.85)	43.89 (16.64 – 99.72)	59.76	35.10	21.22	15.07
Gilan	16.38 (5.20 – 37.55)	28.78 (9.92 – 67.52)	23.97 (8.34 – 53.34)	36.75 (13.39 – 83.88)	29.96 (10.85 – 66.48)	43.18 (16.17 – 98.44)	46.33	27.69	24.98	17.49
Golestan	16.66 (5.29 – 38.36)	29.10 (10.22 – 66.32)	23.87 (8.41 – 52.01)	36.36 (13.27 – 82.91)	29.47 (11.05 – 63.78)	42.44 (16.19 – 97.20)	43.27	24.94	23.46	16.72
Hamadan	14.52 (4.56 – 33.49)	26.46 (9.16 – 61.62)	22.96 (7.98 – 50.32)	35.27 (12.95 – 79.27)	28.96 (10.51 – 63.20)	41.57 (15.59 – 95.25)	58.12	33.29	26.13	17.86
Hormozgan	14.21 (4.26 – 33.38)	26.01 (8.79 – 61.70)	23.00 (7.86 – 51.15)	35.10 (12.51 – 79.50)	28.83 (10.21 – 63.15)	41.30 (15.19 – 95.52)	61.85	34.94	25.34	17.66
Ilam	12.72 (3.67 – 29.86)	24.49 (8.20 – 59.87)	23.78 (8.16 – 52.13)	36.10 (13.62 – 82.70)	30.32 (11.08 – 65.54)	42.96 (16.40 – 97.70)	86.94	47.40	27.50	19.00
Isfahan	17.47 (5.84 – 39.28)	29.01 (10.31 – 67.65)	24.23 (8.80 – 55.92)	37.12 (13.85 – 84.76)	30.71 (11.13 – 66.62)	43.10 (16.61 – 96.91)	38.69	27.95	26.74	16.10
Kerman	17.21 (5.76 – 39.64)	29.36 (10.63 – 67.77)	24.46 (8.48 – 52.49)	36.42 (13.67 – 83.38)	28.58 (10.51 – 62.96)	40.92 (15.44 – 94.14)	42.12	24.04	16.84	12.35
Kermanshah	14.60 (4.70 – 34.91)	26.84 (8.96 – 62.38)	22.22 (7.67 – 49.09)	34.55 (12.61 – 77.67)	28.91 (10.28 – 62.75)	41.59 (15.39 – 95.90)	52.19	28.72	30.10	20.37
North Khorasan	13.33 (3.93 – 30.69)	25.41 (8.68 – 60.63)	21.15 (7.18 – 47.88)	33.76 (12.33 – 77.69)	28.16 (10.25 – 61.81)	40.96 (15.58 – 93.66)	58.66	32.86	33.14	21.32
Khorasan – e – Razavi	14.65 (4.67 – 34.59)	26.83 (9.13 – 62.24)	22.07 (7.32 – 49.63)	34.28 (12.37 – 79.09)	27.33 (9.79 – 58.38)	39.94 (14.93 – 92.74)	50.64	27.76	23.83	16.51
South Khorasan	12.79 (3.71 – 30.61)	24.53 (8.20 – 57.60)	20.54 (6.91 – 46.00)	32.80 (11.91 – 76.15)	27.38 (9.85 – 59.50)	39.77 (14.95 – 88.26)	60.59	33.71	33.30	21.25
Khuzestan	16.13 (5.11 – 37.10)	27.79 (9.68 – 66.40)	24.03 (8.37 – 52.09)	36.09 (13.33 – 83.76)	29.79 (10.99 – 64.14)	42.13 (16.07 – 95.10)	48.97	29.86	23.97	16.73
Kohgiluyeh and Boyer – Ahmad	12.14 (3.43 – 28.63)	23.56 (7.92 – 56.30)	21.29 (7.23 – 47.47)	33.42 (12.20 – 75.63)	29.17 (10.62 – 61.84)	41.63 (15.87 – 93.68)	75.37	41.85	37.01	24.56
Kurdistan	12.50 (3.59 – 29.16)	24.36 (8.17 – 57.51)	20.23 (6.81 – 45.63)	32.53 (11.58 – 74.52)	27.05 (9.55 – 58.85)	39.85 (14.60 – 90.27)	61.84	33.53	33.71	22.50
Lorestan	14.52 (4.48 – 32.30)	26.63 (9.29 – 62.51)	22.87 (8.11 – 49.65)	35.22 (12.97 – 80.85)	29.19 (10.78 – 63.12)	41.96 (15.86 – 94.65)	57.50	27.63	32.25	19.13
Markazi	15.87 (4.95 – 36.18)	27.68 (9.66 – 64.82)	24.00 (8.48 – 52.47)	35.73 (13.28 – 82.43)	29.54 (10.89 – 65.11)	41.80 (15.78 – 96.09)	51.22	29.08	23.08	16.98
Mazandaran	16.81 (5.38 – 37.62)	28.76 (10.20 – 66.84)	25.67 (9.17 – 55.84)	37.80 (13.67 – 86.25)	32.17 (11.74 – 69.69)	44.36 (17.09 – 100.84)	52.70	31.43	25.32	17.35
Qazvin	14.95 (4.67 – 34.19)	26.75 (9.11 – 61.79)	23.34 (8.07 – 51.19)	35.19 (12.86 – 78.26)	29.51 (10.83 – 65.56)	41.94 (15.43 – 93.77)	56.12	31.55	26.43	19.18
Qom	16.53 (5.48 – 36.92)	27.57 (9.60 – 62.78)	24.01 (8.39 – 53.09)	35.37 (12.85 – 81.24)	30.48 (11.42 – 66.55)	42.24 (15.86 – 96.48)	45.24	28.29	26.94	19.42
Semnan	15.16 (4.69 – 35.43)	26.87 (9.38 – 62.73)	24.36 (8.74 – 53.39)	36.40 (13.46 – 84.86)	30.84 (11.18 – 67.55)	43.20 (16.32 – 97.43)	60.68	35.46	26.60	18.68
Sistan and Baluchistan	11.97 (3.49 – 28.10)	21.98 (7.38 – 52.02)	18.09 (6.07 – 41.49)	28.93 (10.35 – 65.17)	23.37 (8.01 – 50.78)	34.76 (12.62 – 80.08)	51.12	31.61	29.18	20.15
Tehran	18.93 (6.34 – 42.94)	31.43 (11.38 – 72.30)	26.68 (9.55 – 58.03)	39.37 (14.86 – 90.41)	32.63 (12.33 – 71.07)	45.54 (17.74 – 102.29)	40.94	25.26	22.30	15.67
Yazd	17.19 (5.51 – 39.22)	29.19 (10.48 – 69.36)	24.76 (8.73 – 54.52)	36.88 (13.59 – 83.78)	31.36 (11.46 – 69.38)	43.75 (16.24 – 98.06)	44.03	26.34	26.65	18.62
Zanjan	14.04 (4.32 – 32.91)	25.68 (8.59 – 60.92)	21.49 (7.54 – 47.99)	33.35 (12.05 – 77.11)	28.13 (10.28 – 63.99)	40.61 (15.52 – 95.38)	53.06	29.86	30.89	21.76
Iran	15.93 (5.15 – 36.82)	28.00 (9.84 – 64.53)	23.96 (8.30 – 52.59)	36.19 (13.48 – 82.79)	29.92 (10.98 – 64.92)	42.50 (16.32 – 97.37)	50.40	29.25	24.87	17.43

In 1990, 2005, and 2019, the DALYs/100,000 attributed to high BMI for OA in Iranian men were 15.93 (95% CI: 5.15–36.82), 23.96 (95% CI: 8.30–52.59), and 29.92 (95% CI: 10.98–64.92), respectively. In Iranian women, the rate of DALYs/100,000 attributable to high BMI for OA were 28.00 (95% CI: 9.84–64.53), 36.19 (95% CI: 13.48–82.79), and 42.50 (95% CI: 16.32–97.37), respectively, in 1990, 2005, and 2019. The highest percentage changes in the rate of DALYs/100,000 attributable to high BMI for OA were observed in Kohgiluyeh and Boyer-Ahmad, North Khorasan, Kurdistan, South Khorasan, and Lorestan provinces for men and in Kohgiluyeh and Boyer-Ahmad and Kurdistan provinces for women. Province-specific DALYs/100,000 for high BMI for OA in men and women are shown in Figs. 1A,B.

**Figure 2:**
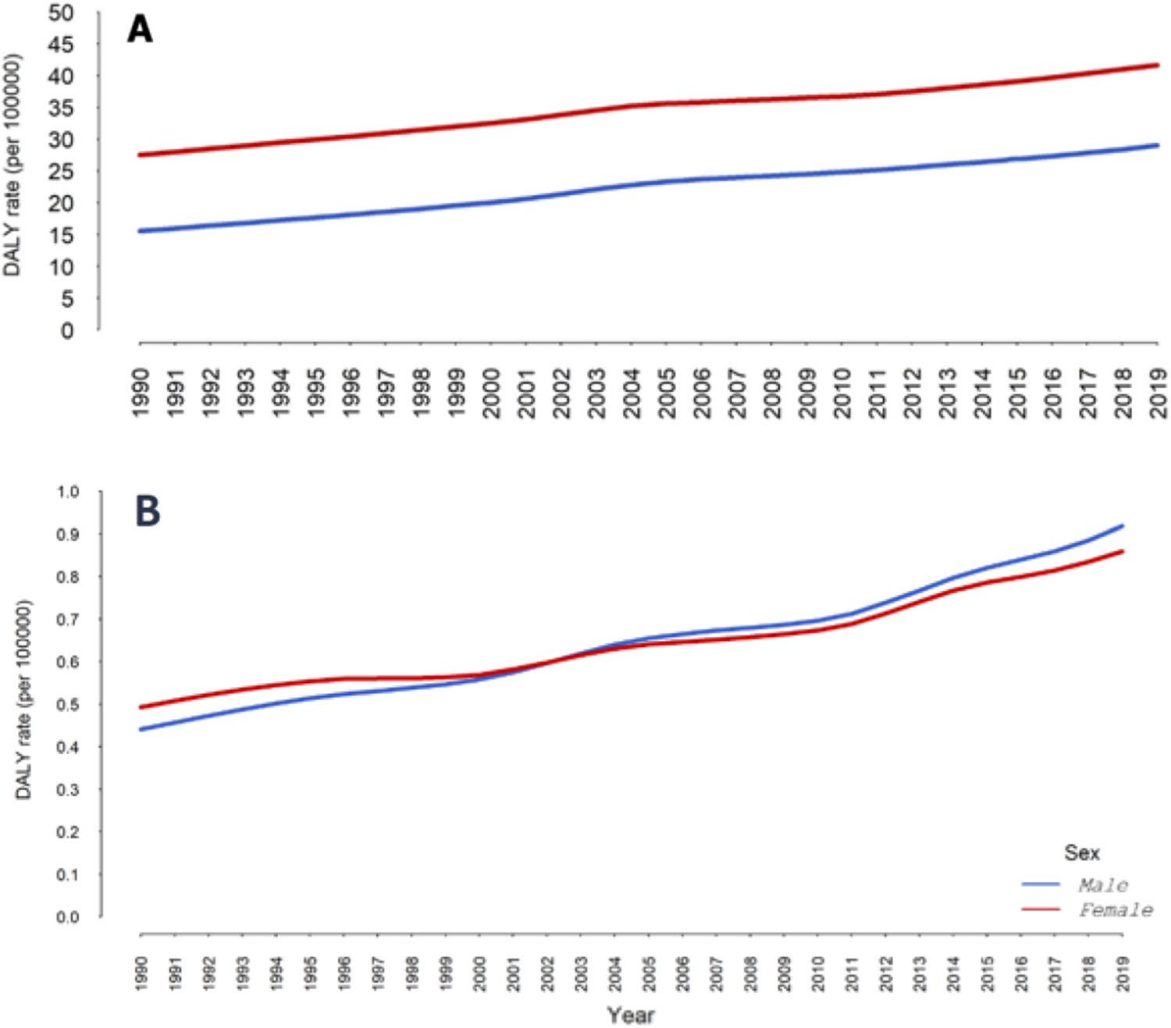
Age-Standardized DALYs rate (per 100,000 population) (**A**) Hip and (**B**) Knee Osteoarthritis attributable to high BMI in Iran between 1990 and 2019.

As shown in Fig. 2, the DALYs/100,000 rates for knee and hip OA attributable to high BMI show an increasing trend for men and women between 1990 and 2019. In addition, the burden of hip OA is dramatically higher than that of knee OA, especially among women.

**Figure 3:**
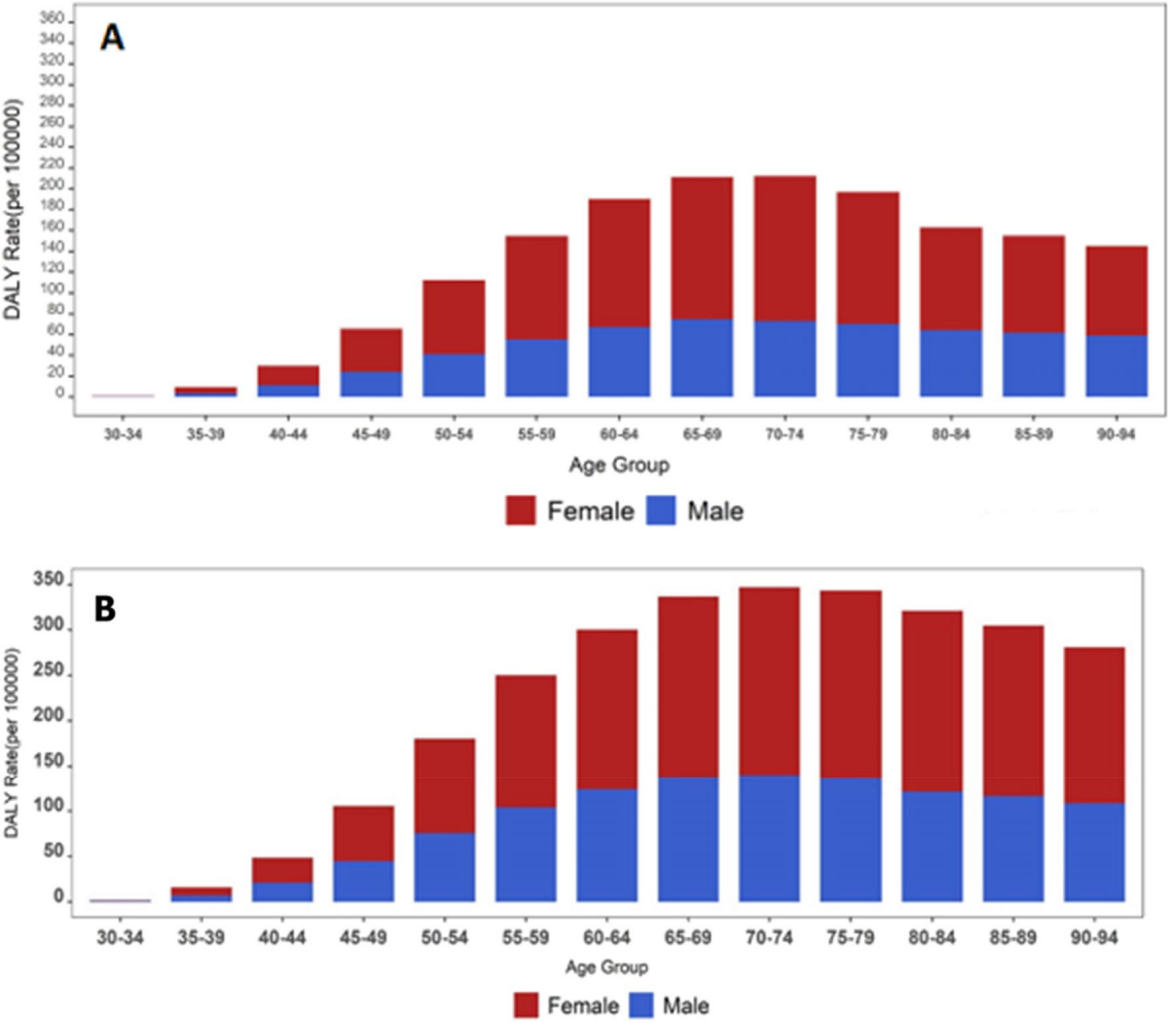
DALYs rate (per 100,000 populations) (**A**) 1990 and (**B**) 2019 osteoarthritis attributable to high BMI in Iran by age groups.

The highest DALYs/100,000 rate for OA attributed to high BMI was in women and men 65 to 69 and 75 to 79 years old in 1990; and in 2019 DALYs is increased in all age groups; with the highest DALYs/100,000 rates in 2019 in 65 to 79-year age group (Fig. 3). Stacked figures show the DALYs/100,000 rate for OA attributed to high BMI by sex and age groups in 31 provinces of Iran from 1990 to 2019.

**Figure 4:**
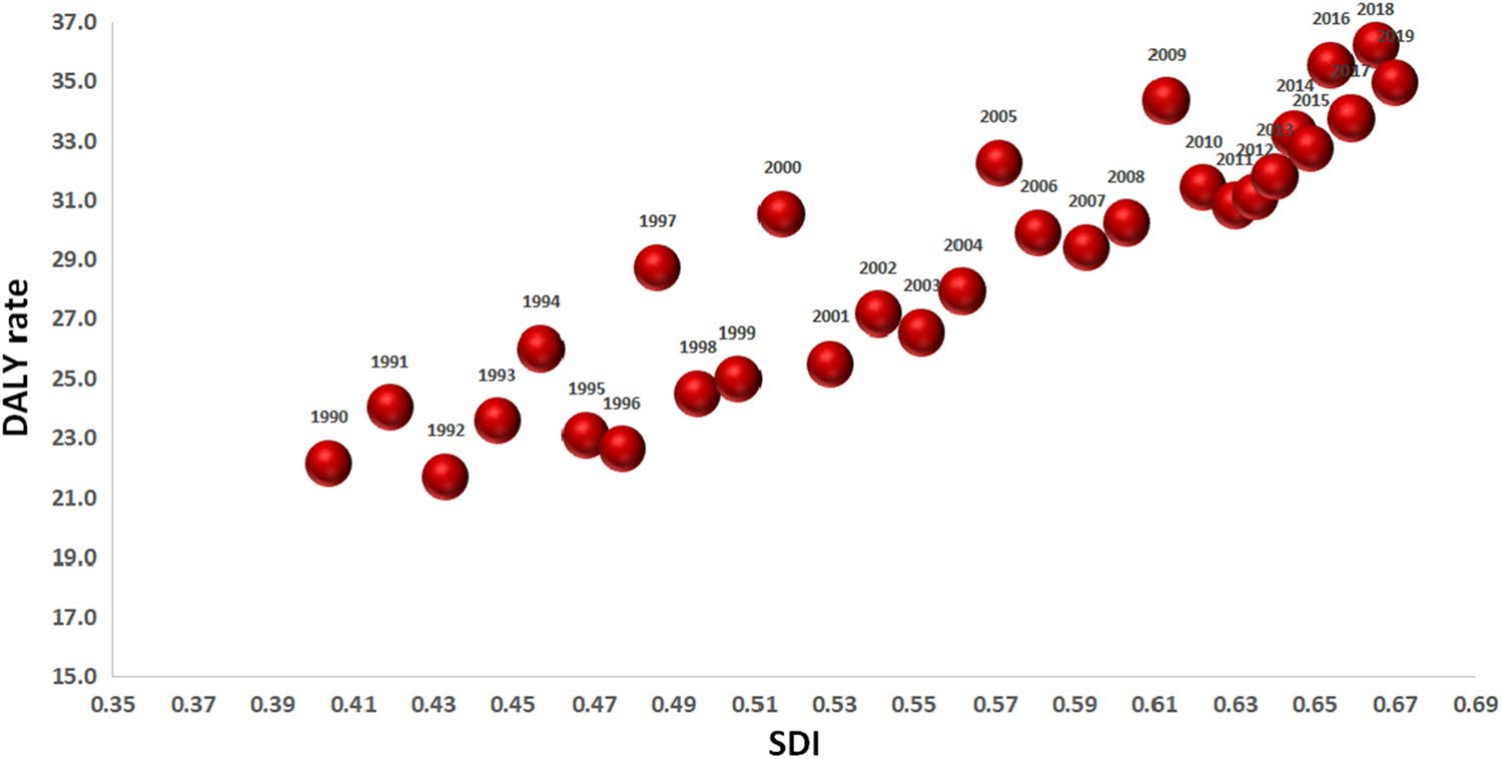
DALYs rate (per 100,000 populations) osteoarthritis attributable to high BMI by Socio-demographic Index (SDI) from 1990 to 2019 in Iran.

The Fig. 4 has shown that the age-standardized DALYs rate of OA due to high BMI were positively associated with the Socio-demographic Index (SDI) in Iran population, and this association has gradually become stronger over the last three decades.

## Discussion

Consistent to GBD 2019 study, we comprehensively assessed the epidemiological trends of the prevalence and burden of OA due to high BMI from 1990 to 2019 in Iran. In general, the results of this study show that the prevalence of OA has increased dramatically over the past three decades in Iran. The burden of knee and hip OA attributed to high BMI has increased significantly in all age groups in both Iranian men and women from 1990 to 2019. Between 2005 and 2019, the rate of DALYs/100,000 related to high BMI for OA in men and women changed by 24.87 and 17.43 percents, respectively.

Studies in other regions have also found an increase in the prevalence of OA^[3,20,21]^. The prevalence of OA has increased from 8.2% in 1998 to 10.7% in 2017 in both men and women in the United Kingdom^[20]^. The prevalence of OA increased by approximately 43% between 1990 and 2015 in the Nordic region^[3]^. In addition, OA caused 52,661 YLDs in 2015 and was identified as the 15th leading cause of YLDs in this region and the 8th leading cause of YLDs in women aged 65–74 years. Notably, 23% of YLDs due to OA are attributable to high BMI^[3]^. A systematic review study by Liu et al., showed that the burden of knee and hip OA due to high BMI increased significantly in China and the United States from 1990 to 2019^[4]^. Results from a large longitudinal cohort also showed a positive association between obesity and the risk of knee OA^[22]^. This association is also supported by other studies, indicating that the association becomes stronger with increasing BMI^[6,23–25]^. The increase in overweight, obesity and the aging of the population in Iran are possible explanations for the increasing trend of OA due to high BMI. Furthermore, lifestyle changes, such as reduction in physical activity and a high in fat diet with refined carbohydrates and high calories, may also be the causes of the increasing trend of OA due to obesity. The limited effect of OA treatments is also critical to the increase in prevalence and DALYs. Moreover, the role of diagnostic and medical advances, which have led to the identification of patients and reporting of more accurate cases, should not be ignored.

Consistent with previous studies, age and female sex are the strongest risk factors for OA^[15,26]^. The DALYs rate for OA attributed to high BMI has recently increased in all age groups, and the highest DALYs/100,000 rate was observed between 65 and 79 years. In other studies, the age group of 65 to 79 years is the most affected age group by OA due to obesity^[3,4]^. The findings of this study show that the DALYs rate for knee and hip OA attributed to high BMI in Iran and its 31 provinces is significantly higher in women than in men. However, the percentage change was higher in men than in women. Studies from China, the United States, and the Nordic region also indicate the DALYs rate for OA due to high BMI to be higher in women than in men^[3,4]^. A population-based cohort study found that the risk of knee and hip OA due to high BMI in women were respectively 53% (Hazard ratio: 1.53) and 20% (Hazard ratio: 1.20) higher than in men ^[11]^. In the study by Misra et al., the relative risk (RR) for OA was 2.29 and 1.73 times higher in obese women and men, respectively, then in normal-weight women and men^[22]^.

The results confirm that the DALYs rate attributable to high BMI is dramatically higher for OA at the hip than for OA at the knee, especially in women, and their trend is increasing in both sexes. Previous studies have reported the highest incidence, prevalence, and disability for hip OA to occur in women and obese individuals^[27,28]^. A meta-analysis study in 2023 reported the prevalence of hip OA to be 8.55% (1.2–12.6%), showing an increasing trend with age^[29]^. In contrast, a meta-analysis in 2019 found that the prevalence of asymptomatic knee OA in adults aged < 40 years was 4–14% and in adults ≥ 40 years was 19–43%^[30]^. The study by Singer et al. showed that increasing BMI was associated with increasing knee and hip OA^[31]^. These findings highlight the importance of regular screening for OA and early diagnosis of those at risk.

The present study showed that age-standardized DALYs rate of OA due to high BMI were positively associated with the SDI in Iran population, and this association has gradually become stronger over the last three decades. On the other hand, analysis of the distribution among provinces in this study showed that the prevalence of OA is higher in the north–south belt. This finding may be due to differences in socio-economic status, geography and access to healthcare services. Tehran, Shiraz, and Isfahan provinces (north–south belt) are cities with higher income levels. Regional and global surveys have also shown that symptomatic hip OA is higher in high-income countries than in low-income countries^[32]^. Interestingly, previous studies have reported that socio-economic status has a significant association with OA^[32,33]^. In addition to the study by Witkam et al., BMI has been introduced as a mediating variable in the relationship between socio-economic status and OA^[33]^.

The trend toward OA is expected to continue to increase, especially in the coming years, due to rising obesity, increasing life expectancy, and the country’s aging population. Overweight and obesity increase the risk of OA through two possible mechanisms of increasing the mechanical load on the joints and causing inflammation^[13,34,35]^. According to the study by Reyes et al., the incidence of knee OA in people with grade II obesity was 19.5 and in normal-weight people it was 3.7 per 1,000 persons-year^[11]^; however, compared with other risk factors for OA, such as age, sex, and joint damage, high BMI can be considered a modifiable risk factor. Therefore, it is necessary to provide preventive solutions at the individual and social levels. At the population level, it is recommended to increase public awareness of the importance of a healthy lifestyle, including age-appropriate exercise, weight control, balanced diet, instruction in proper sitting and standing by ergonomic experts, and improvement of workplace safety and ergonomics. At the individual level, it is recommended that patients be better educated about the nature and treatment of OA and the importance of self-care in this disease, as well as regular physical activity, weight control, and weight loss.

This study provides comprehensive information on the burden of OA due to high BMI in Iran and in each of the 31 provinces. However, this study has limitations. The GBD data were not original and acquired based on models. However, the GBD uses standard instruments to improve the accuracy of the data. In addition, the burden of OA was limited to knee and hip OA and did not include other sites. This limitation may have resulted in an underestimation of the burden of OA in this study. Nevertheless, these results present an up-to-date assessment of the burden of OA in 31 Iranian provinces and provide valuable information for policymakers and health professionals. It is suggested that controlled clinical trial studies (RCT) be conducted to reduce the weight of new cases of OA. Also, studies that examine urban and rural populations and individuals under 30 years of age may shed some light on the subject.

## Conclusion

The burden of knee and hip OA due to high BMI has recently increased significantly in Iran in all age groups and in both sexes. Also, with increase in population age and obesity, the burden of OA is expected to increase substantially. Burden of OA due to high BMI were positively associated with the Socio-demographic Index (SDI). It is recommended that health policymakers develop and implement weight control strategies at the national level to reduce overweight, obesity and burden of OA.

## Data Availability

The data of this study obtained from the Global Health Data Exchange query tool (https://vizhub.healthdata.org/gbd-compare/). Access to the data of the present study through this site is free for all researchers.
